# Multiple Cardiometabolic Risk Factors in the Southern Cone of Latin America: A Population-based Study in Argentina, Chile, and Uruguay

**DOI:** 10.1016/j.ijcard.2015.01.062

**Published:** 2015-01-27

**Authors:** Adolfo L. Rubinstein, Vilma E. Irazola, Matias Calandrelli, Natalia Elorriaga, Laura Gutierrez, Fernando Lanas, Jose A. Manfredi, Nora Mores, Hector Olivera, Rosana Poggio, Jacqueline Ponzo, Pamela Seron, Chung-Shiuan Chen, Lydia A. Bazzano, Jiang He

**Affiliations:** a Southern Cone American Center of Excellence for Cardiovascular Health, Institute for Clinical Effectiveness and Health Policy, Buenos Aires, Argentina; b Argentina Sanatorio San Carlos, Bariloche, Pcia de Río Negro, Argentina; c Universidad de La Frontera, CIGES, Temuco, Chile; d Faculty of Medicine, Universidad de la República, Montevideo, Uruguay; e Municipalidad de Marcos Paz, Pcia de Buenos Aires, Argentina; f Department of Epidemiology, Tulane University School of Public Health and Tropical Medicine, New Orleans, LA, USA

**Keywords:** cardiovascular disease, diabetes, dyslipidemia, hypertension, obesity, risk factors

## Abstract

**Background:**

Cardiovascular disease is a major cause of death, and its mortality is increasing in Latin America. However, population-based data on cardiovascular disease risk factors are sparse in these countries.

**Methods:**

A total of 7,524 men and women, aged 35 to 74 years old, were recruited between February 2010 and December 2011 from randomly selected samples in 4 cities (Bariloche and Marcos Paz, Argentina; Temuco, Chile; and Pando-Barros Blancos, Uruguay) in the Southern Cone of Latin America. Cardiovascular risk factors were measured using standard methods by trained and certified observers.

**Results:**

Approximately 85.5% of adults ate less than five servings of fruit or vegetables per day, 35.2% engaged in low physical activity, and 29.7% currently smoked cigarettes. The prevalences of obesity, central obesity, hypertension, chronic kidney disease, dyslipidemia, diabetes, and metabolic syndrome were 35.7%, 52.9%, 40.8%, 2.0%, 58.4%, 12.4%, and 37.4%, respectively. The proportion of individuals with ≥3 cardiovascular risk factors, including low intake of fruit and vegetables, low physical activity, current cigarette smoking, obesity or central obesity, hypertension, chronic kidney disease, dyslipidemia, and diabetes, was 68.3%, and the proportion of individual with ≥3 cardiometabolic risk factors, including obesity or central obesity, hypertension, chronic kidney disease, dyslipidemia, and diabetes, was 22.9%.

**Conclusions:**

Cardiovascular disease risk factors are highly prevalent in the general population in the Southern Cone of Latin America. These data suggest that national efforts on the prevention, treatment, and control of cardiovascular risk factors should be a public health priority in the Southern Cone of Latin America.

## Introduction

Coronary heart disease (CHD) and stroke are the leading causes of deaths worldwide, collectively killing 12.9 million people in 2010, or one in four deaths (1). Low- and middle-income countries (LMIC) are disproportionally affected: over 80% of cardiovascular disease (CVD) deaths occur in LMIC, and almost half of CVD deaths are in people younger than 70 years in these countries (2). It is estimated that the number of CVD deaths in Latin America will increase by more than 60% between 2000 and 2020 while CVD deaths will increase by only 5% in high-income countries during the same period (3). In a case-control study of 1,237 CHD patients and 1,888 controls in South America, abdominal obesity, dyslipidemia, cigarette smoking, and hypertension were associated with high population-attributable risks of 48.5%, 40.8%, 38.4%, and 32.9%, respectively (4). These risk factors jointly accounted for 88% of the population-attributable risk. However, population-based data on the prevalence of these risk factors are sparse in Argentina, Chile and Uruguay. Furthermore, the limited available information on CVD risk factors in these populations was predominantly from studies based on self-reported data (5,6) or conducted in small convenience samples (7). Self-reported CVD risk factors do not provide reliable estimates of disease burden because they are influenced by access to healthcare and other factors (9).

The CESCAS (Centro de Excelencia en Salud Cardiovascular para el Cono Sur) I study is a population-based study aimed to examine CVD and risk factors in the general population from four representative cities in the Southern Cone of Latin America (10). Specifically, the objectives of the present analysis are to provide current and reliable data on population levels of behavioral and metabolic risk factors for CVD and to assess the distribution of multiple CVD risk factors in the general adult population in the Southern Cone of Latin America.

## Methods

### Study participants

The details of study design and sampling method of the CESCAS I study have been published earlier (9). Briefly, 7,524 women and men, aged 35 to 74 years old, were recruited between February 2010 and December 2011 from randomly selected samples in 4 small to mid-sized cities in the Southern Cone of Latin America: two cities located in Argentina (Bariloche and Marcos Paz), one in Chile (Temuco), and one in Uruguay (Pando-Barros Blancos). Marcos Paz and Pando-Barros Blancos are small cities with 54,000 and 58,000 residents, respectively, according to the latest census data. Both urban and rural participants were recruited from these sites. Bariloche (Argentina) and Temuco (Chile) are larger cities with 134,000 and 245,000 residents, respectively, according to the latest census data. These study locations were selected based on population characteristics reflecting country averages. In addition, all four locations have demonstrated stable populations with migration rates below 10% over the past 10 years.

A 4-stage stratified sampling method was used to select a representative sample of the general population of the Southern Cone of Latin America (10). In the first stage, census radii were randomly selected from each of the four locations, stratified by socio-economic level. In the second stage, a number of blocks proportional to the radius size were randomly selected. In the third stage, households within each block were selected by systematic random sampling. All members between 35-74 years in the selected households were listed to create the study sampling frame. In the final stage of sampling, one listed member per household was randomly selected to be included in the study.

Of the 10,254 individuals randomly selected, 550 were never found at their homes and 1,394 refused to participate. Of those 8,310 who completed the home surveys, 855 did not attend the clinical examination. Thus, the final sample for this analysis includes 7,524 participants (3,165 men and 4,359 women). The overall response rate was 73.4% and the response rates were similar in men and women and across different locations.

The study complies with the Declaration of Helsinki. The study protocol has been approved by IRBs in all participating institutes in Argentina, Chile, Uruguay and the US. The written informed consent has been obtained from all study participants.

### Data collection

Study data were collected at a home visit and a clinical visit. During the home survey, information on demographic characteristics, including age, sex, education, occupation, household income, and healthcare access; personal history of CVD and risk factors, including CHD, stroke, hypertension, diabetes, and dyslipidemia; treatment of hypertension, diabetes, and dyslipidemia; lifestyle risk factors, including cigarette smoking, alcohol consumption, and physical activity; and diet was obtained using a standard questionnaire. Leisure time physical activity, domestic and gardening activities, work-related physical activity, and transport-related physical activity were obtained using the International Physical Activity Questionnaire-Short Form (11). The recorded activities were converted into metabolic equivalent (MET) and low activity was defined as <600 MET-minutes/week of total physical activity (12). Nutrition information was collected using a semi-quantitative, self-administered food frequency questionnaire adapted from the NCI Dietary History Questionnaire and validated in Argentina, Chile, and Uruguay (13,14). Specifically, the list of foods and beverages was modified to include those frequently consumed in Argentina, Chile and Uruguay according to data obtained from national surveys or food lists included in other food frequency questionnaires already validated in these countries (15-19). Low fruit and vegetable intake was defined as <5 servings per day.

During the clinical examination, blood pressure (BP) and anthropometric measurements were obtained by trained and certified observers using standard protocols and techniques (20). Three BP measurements were obtained with the participant in the seated position after 5 minutes of rest using a standard mercury or aneroid sphygmomanometer, and the mean of three readings was used for analysis. Participants were advised to avoid cigarette smoking, alcohol, caffeinated beverages, and exercise for at least 30 min before their BP measurement. Body weight, height, and waist circumference were measured twice during the examination. Weight was measured in light indoor clothing without shoes in kilograms to one decimal place, using standing scales supported on a steady surface. Height was measured without shoes in centimeters to one decimal place with a stadiometer. Waist circumference was measured at 1 cm above the navel at minimal respiration in centimeters to one decimal place.

Overnight fasting blood specimens were obtained for measurement of lipids, creatinine, and glucose. The fasting time was verified before the blood specimen was taken. Participants who had not fasted for at least 10 hours did not have their blood drawn. Blood specimens were processed at the examination center and shipped to a central clinical laboratory in Buenos Aires where the specimens were stored at −80°C until laboratory assays could be done. Blood glucose, total cholesterol, HDL-cholesterol, triglycerides, and creatinine were measured using standard methods with commercially available reagents. LDL-cholesterol was calculated using the Friedewald equation for participants with triglycerides <400 mg/dL (21).

Hypertension was defined as mean systolic BP ≥140 mm Hg, and/or diastolic BP ≥90 mm Hg, and/or current use of antihypertensive medications. Obesity was defined as a body-mass index (BMI) ≥30 kg/m^2^ and overweight as BMI ≥25 and <30 kg/m^2^. Central obesity was defined as waist circumference ≥102 cm for men or ≥88 cm for women (22). Dyslipidemia was defined as total cholesterol ≥240 mg/dL and/or LDL-cholesterol ≥160 mg/dL and/or HDL-cholesterol <40 mg/dL and/or triglyceride ≥200 mg/dL and/or use of lipid-lowering medication. Diabetes was defined as fasting glucose ≥126 mg/dL or self- reported history of diabetes (23). Metabolic syndrome was defined as 3 or more metabolic risk factors: waist circumference ≥102 cm in men and 88 cm in women, triglyceride ≥150 mg/dL, HDL-cholesterol <40 mg/dL in men and <50 mg/dL in women, blood pressure ≥135/85 mm Hg or use of antihypertensive medications, and fasting glucose ≥110 mg/dL or anti-diabetic therapy (22).

### Statistical analysis

The CESCAS I study was designed to provide precise estimates of the prevalence of CVD risk factors by sex and region (Marcos Paz and Bariloche, Argentina; Temuco, Chile; and Pando-Barros Blancos, Uruguay) in four age groups: 35-44, 45-54, 55-64 and 65-74 years old. Sample sizes were estimated to meet generally recommended requirements for precision in a complex survey (24). All calculations were weighted to represent the general adult population aged 35-74 years in the study sites. Weights were calculated on the basis of data from the 2010 Population Census and the CESCAS I study sampling scheme, and took into account several features of the survey, including oversampling for specific age groups, non-response, and other demographic differences between the sample and the total population.

Mean level and prevalence estimates of CVD risk factors were calculated for the overall population and by the four age groups. Additionally, age-standardized prevalence estimates were calculated for men and women, and the four study sites, after age-standardization to the overall 2010 population distribution in the Southern Cone of Latin America. Standard errors were calculated by a technique appropriate for the complex survey design. All data analyses were done with SUDAAN (Version 10.0; Research Triangle Institute, Research Triangle Park, NC, USA) and STATA 12.0 (StataCorp LP, College Station, TX, USA).

## Results

### Demographic and behavioral risk factors

The demographic and behavioral risk factors in the general population aged 35-74 years in the Southern Cone of Latin America are presented in table 1. Approximately 52.3% individuals did not graduate from high school (52.4% in men and 52.2% in women), 6.0% were unemployed (5.1% in men and 6.8% in women), and 56.2% did not report to have social security or private health insurance (54.8% in men and 57.4% in women).

Approximately 85.5% of adults (89.8% men and 81.7% women) aged 35-74 years in the Southern Cone ate less than five servings of fruit or vegetables per day. The low intake of fruit and vegetables was consistent across various regions and age groups. About 35.2% of adults (28.3% men and 41.3% women) in the Southern Cone had low physical activity, which varied by region and increased with age. In addition, 29.7% of adults (33.3% men and 26.5% women) in the Southern Cone were current cigarette smokers. The prevalence of cigarette smoking was consistent among the four study regions and decreased with age.

### Metabolic risk factors

Mean BMI and waist circumference were 28.9 kg/m^2^ and 96.5 cm, respectively, among adults aged 35-74 years in the Southern Cone (table 2). Women had higher BMI while men had higher waist circumference. BMI and waist circumference varied among geographic regions and increased with age. Mean systolic and diastolic BP were 127.1 and 82.3 mm Hg, respectively, and both were higher in men than in women. Systolic BP increased with age for the entire lifespan, but diastolic BP increased with age until 64 years, after which it decreased. Mean serum total, LDL- and HDL-cholesterol, and triglyceride were 201.7, 126.2, 45.7, and 158.8 mg/dL, respectively. HDL-cholesterol was higher in women while triglyceride was higher in men. In general, serum lipids increased with age until 64 years, and then decreased, except for HDL-cholesterol, which increased over the entire lifespan. Mean fasting plasma glucose was 98.0 mg/dL, higher in men than in women, and increased with age. Mean eGFR was 97.7 mil/min/1.73m^2^, higher in women than in men, and decreased with age.

Table 3 shows the prevalence of cardiometabolic risk factors among adults aged 35-74 years in the Southern Cone. The prevalences of overweight, obesity, and central obesity were 41.3%, 35.7%, and 52.9%, respectively. The prevalences of obesity and central obesity were higher in women while overweight was higher in men. The prevalence of hypertension was 40.8% (44.7% in men and 37.3% in women), chronic kidney disease (CKD) 2.0% (1.8% in men and 2.2% in women), dyslipidemia 58.4% (68.3% in men and 49.6% in women), diabetes 12.4% (10.6% in men and 14.0% in women), and metabolic syndrome 37.4% (34.5% in men and 40.0% in women). In general, cardiometabolic risk factors increased with age, except for overweight and hypertriglyceridemia. The prevalence of low HDL-cholesterol decreased with age.

### Multiple cardiovascular risk factors

**Figure 1:**
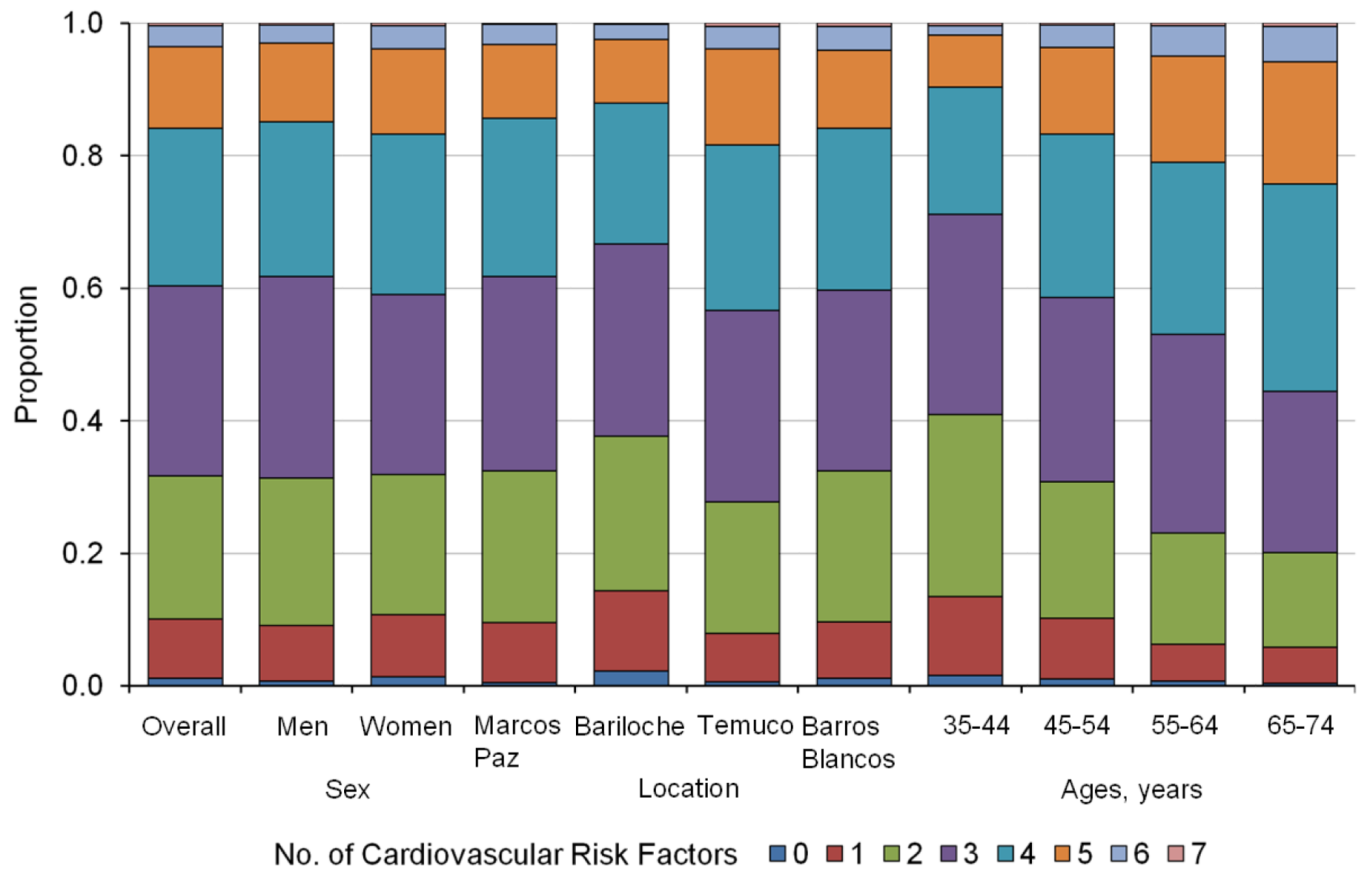
Proportions of the population with multiple behavioral and metabolic risk factors for cardiovascular disease in the Southern Cone of Latin America. Risk factors include low intake of fruit and vegetables, low physical activity, current cigarette smoking, obesity or central obesity, hypertension, chronic kidney disease, dyslipidemia, and diabetes.

**Figure 2:**
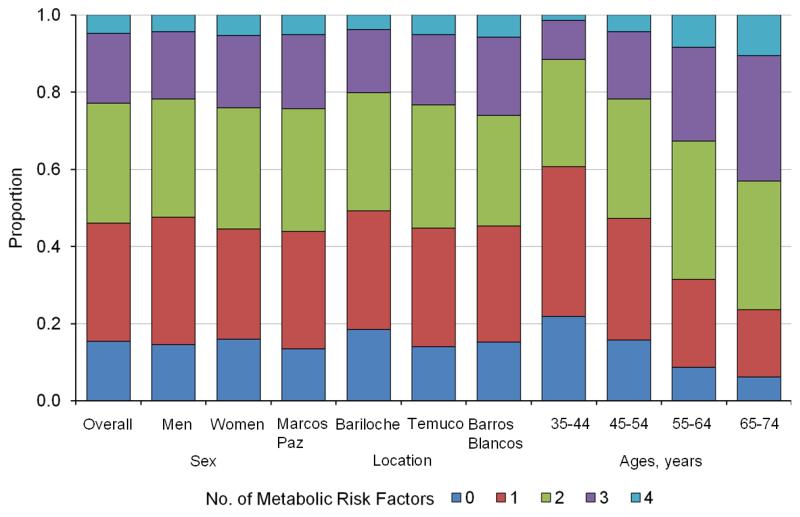
Proportions of the population with multiple cardiometabolic risk factors in the Southern Cone of Latin America. Cardiometabolic risk factors include obesity or central obesity, hypertension, chronic kidney disease, dyslipidemia, and diabetes.

Figure 1 shows the proportions of individuals with multiple CVD risk factors, including low intake of fruit and vegetables, low physical activity, current cigarette smoking, obesity or central obesity, hypertension, CKD, dyslipidemia, and diabetes. Overall, the proportions of individuals with 0, 1, 2, 3, 4, 5, 6 and 7 risk factors were 1.1%, 9.0%, 21.6%, 28.7%, 23.7%, 12.4%, 3.1%, and 0.4%, respectively. The proportions of individuals with ≥3 risk factors were similar in men and women (68.5% vs. 68.1%) while the proportions of individuals with ≥5 risk factors were lower in men than in women (14.9% vs. 16.8%). The number of CVD risk factors increased with age. For example, the proportions of individuals with ≥3 risk factors were 9.7%, 16.8%, 21.0%, and 24.3% among individuals aged 35-44, 45-54, 55-64, and 65-74 years. The proportions of individuals with multiple cardiometabolic risk factors, including obesity or central obesity, hypertension, CKD, dyslipidemia, and diabetes, are displayed in figure 2. Overall, 15.4%, 30.7%, 31.0%, 18.1%, and 4.8% of individuals had 0, 1, 2, 3, and 4 cardiometabolic risk factors. The proportions of individuals with ≥3 cardiometabolic risk factors were higher in women (24.0%) than in men (21.7%) and increased with age: 11.5%, 21.8%, 32.7%, and 43.0% for those aged 35-44, 45-54, 55-64, and 65-74 years, respectively.

## Discussion

Our study indicates that behavioral and metabolic risk factors for CVD are high in the general adult population of the Southern Cone of Latin America. For example, 68.3% of individuals have three or more risk factors, including low intake of fruit and vegetables, low physical activity, current cigarette smoking, obesity or central obesity, hypertension, CKD, dyslipidemia, and diabetes. Furthermore, 77.0% of individuals are overweight or obese, 52.9% have central obesity, 40.8% have hypertension, 58.4% have dyslipidemia, 12.4% have diabetes, and 37.4% have metabolic syndrome. The findings from our study and others warrant an epidemic of cardiovascular diseases in the near future in Latin America without effective intervention (25,26).

These data have important public health implications and should provide evidence for health policy change. Our data show that over 85% of adults consume less than five servings of fruit and vegetables per day, which is recommended for a healthy diet (27). In addition, over 35% of adults have low physical activity and almost 30% of adults are current cigarette smokers. Unhealthy diet, physical inactivity, and cigarette smoking are major lifestyle risk factors for CVD and its risk factors (28). National programs which focus on lifestyle modification interventions should be initiated or strengthened in the Southern Cone of Latin America to combat the epidemic of CVD and risk factors (29).

One of the most striking findings in our study is the very high prevalence of overweight, obesity, and central obesity in the study population. Overweight and obesity affected 3 out of 4 adults and central obesity affected half of adults in the Southern Cone of Latin America. Obesity is an important risk factor for hypertension, dyslipidemia, diabetes, and metabolic syndrome, which were all high in the study population. Therefore, the prevention and control of obesity should be a public health priority in the Southern Cone of Latin America.

A high prevalence of obesity and CVD risk factors was reported in Latinos living in the US (30). The Hispanic Community Health Study/Study of Latinos (HCHS/SOL) reported 36.5% male and 42.6% female Latinos aged 18-74 years in the US were obese. The prevalence of cigarette smoking and hypertension in the Southern Cone population was higher than in the US while the prevalence of diabetes was higher in US Latinos. However, diabetes was defined as a fasting plasma glucose ≥126 mg/dL, 2-hour postload plasma glucose ≥200 mg/dL, an HbA1c ≥6.5%, or use of antihyperglycemic medications in HCHS/SOL (30). Our study suggests that Latinos living in the Southern Cone have similar CVD risk compared to those living in the US.

The Cardiovascular Risk Factor Multiple Evaluation in Latin America (CARMELA) study examined CVD risk factors among individuals living in seven major cities in Latin American countries, including Buenos Aires and Santiago, the capitals of Argentina and Chile, respectively (31). As compared to CARMELA, our study reported a higher prevalence of hypertension, diabetes and obesity. If the comparisons are restricted to only Buenos Aires and Santiago, these differences are more striking. The differences in CVD risk factors between CARMELA and ours might partially reflect the secular increases in CVD risk factors in the Southern Cone, since measurements in CARMELA were performed in 2005, as compared to ours in 2011-12.

Our study is one of the first studies in Latin America to systematically examine CVD risk factors in a representative sample of a general population. Physical and biochemical risk factors were measured using standard methods, which can reduce potential bias due to self-reported data. In addition, a stringent quality control procedure was implemented in all stages of the study. This study provides the most recent and reliable data on CVD risk factors in the general adult population in the Southern Cone of Latin America. Several cohort studies reported that Hispanics had lower cardiovascular disease and all-cause mortality compared to non-Hispanic Whites, despite higher prevalence of cardiovascular disease risk factors and lower socioeconomic status, a phenomenon called the Hispanic paradox (32,33). Future investigations into the role of lifestyle risk factors and genetic predisposition on cardiovascular disease risk in this cohort could help to identify protective factors against cardiovascular disease in Hispanics.

In conclusion, our study indicates that CVD risk factors are highly prevalent in the general population in the Southern Cone of Latin America. The prevalence of CVD risk factors in this population is higher than in other LMIC and high-income countries (34,35). These data suggest that national efforts on the prevention, treatment, and control of CVD risk factors should be a public health priority in the Southern Cone of Latin America.

## Figures and Tables

**Table 1:** General Characteristics and Behavioral Risk Factors of the Study Population in the Southern Cone of Latin America

	No. of Study Participants	Less than High School, %	Unemployed, %	No Health Insurance*, %	Low Intake of Fruit and Vegetables^†^, %	Low Physical Activity^†^, %	Current Cigarette Smoking, %
Overall	7,524	52.3 (50.9, 53.7)	6.0 (5.3, 6.7)	56.2 (54.8, 57.6)	85.5 (84.6, 86.5)	35.2 (33.8, 36.5)	29.7 (28.4, 31.0)

Sex							
Men	3,165	52.4 (50.3, 54.5)	5.1 ( 4.2, 6.1)	54.8 (52.7, 56.8)	89.8 (88.6, 91.0)	28.3 (26.4, 30.2)	33.3 (31.3, 35.3)
Women	4,359	52.2 (50.4, 54.1)	6.8 ( 5.7, 7.8)	57.4 (55.6, 59.3)	81.7 (80.3, 83.1)	41.3 (39.4, 43.1)	26.5 (24.8, 28.3)

Location							
Marcos Paz, Argentina	1,991	77.0 (74.9, 79.1)	8.5 ( 7.1, 9.9)	67.5 (65.3, 69.8)	88.8 (87.3, 90.3)	24.3 (22.4, 26.3)	28.7 (26.5, 31.0)
Bariloche, Argentina	1,999	61.5 (59.2, 63.8)	3.4 ( 2.5, 4.3)	26.2 (24.0, 28.3)	82.0 (80.3, 83.8)	27.8 (25.7, 29.8)	28.2 (26.0, 30.4)
Temuco, Chile	1,950	34.9 (32.7, 37.1)	7.3 ( 5.9, 8.6)	74.5 (72.4, 76.6)	87.8 (86.3, 89.3)	42.0 (39.6, 44.4)	30.9 (28.6, 33.2)
Barros Blancos, Uruguay	1,584	73.8 (71.5, 76.1)	5.1 ( 3.9, 6.3)	44.5 (41.8, 47.1)	82.8 (80.9, 84.7)	34.3 (31.9, 36.8)	29.6 (27.2, 32.0)

Age groups, year							
35-44	1,716	40.8 (38.2, 43.4)	8.7 ( 7.2, 10.3)	56.7 (54.0, 59.5)	88.0 (86.3, 89.7)	32.3 (29.6, 34.9)	33.9 (31.2, 36.5)
45-54	2,072	52.0 (49.5, 54.5)	6.4 ( 5.2, 7.6)	59.3 (56.9, 61.7)	87.2 (85.6, 88.8)	34.5 (32.1, 36.8)	35.8 (33.4, 38.1)
55-64	2,114	61.6 (59.2, 64.0)	3.8 ( 2.9, 4.8)	54.5 (52.1, 56.9)	82.8 (80.9, 84.6)	35.6 (33.3, 37.9)	24.2 (22.2, 26.3)
65-74	1,622	71.9 (69.4, 74.4)	0.5 ( 0.1, 0.8)	50.1 (47.4, 52.9)	79.0 (76.8, 81.1)	44.6 (41.9, 47.3)	12.6 (10.9, 14.4)

* Health Insurance only included social security or private insurance.

† Low fruit and vegetable intake was defined as <5 servings per day and low physical activity was defined as <600 MET-minutes/per

**Table 2:** Mean Cardiometabolic Risk Factors in the Southern Cone of Latin America

	Body- mass Index, kg/m^2^	Waist Circumference, cm	Blood Pressure, mm Hg		Serum Cholesterol, mg/dL			Triglyceride, mg/dL	Fasting Plasma Glucose, mg/dL	eGFR, ml/min
	
			Systolic	Diastolic	Total	LDL	HDL			
Overall	28.9 (28.7, 29.0)	96.5 (96.1, 96.8)	127.1 (126.6, 127.6)	82.3 (82.0, 82.7)	201.7 (200.6, 202.9)	126.2 (125.2, 127.1)	45.7 (45.3, 46.0)	158.8 (154.8, 162.7)	98.0 (97.3, 98.8)	97.7 (97.3, 98.1)

Sex										
Men	28.5 (28.3, 28.7)	98.8 (98.3, 99.3)	129.8 (129.1, 130.5)	84.7 (84.2, 85.1)	201.7 (200.0, 203.4)	126.5 (125.1, 127.9)	42.3 (41.8, 42.8)	181.1 (174.0, 188.3)	99.8 (98.7, 100.8)	96.7 (96.1, 97.3)
Women	29.2 (29.0, 29.4)	94.4 (93.9, 94.9)	124.6 (123.9, 125.3)	80.2 (79.8, 80.7)	201.7 (200.2, 203.3)	125.9 (124.6, 127.2)	48.7 (48.3, 49.2)	138.7 (135.1, 142.3)	96.5 (95.4, 97.6)	98.6 (98.1, 99.2)

Location										
Marcos Paz, Argentina	30.0 (29.7, 30.2)	97.8 (97.1, 98.5)	128.0 (127.1, 129.0)	81.0 (80.5, 81.6)	202.7 (200.6, 204.8)	127.6 (125.8, 129.4)	44.7 (44.1, 45.3)	159.5 (153.4, 165.7)	101.7 (100.1, 103.4)	98.4 (97.6, 99.2)
Bariloche, Argentina	28.4 (28.1, 28.6)	94.6 (94.0, 95.3)	127.3 (126.5, 128.1)	85.3 (84.8, 85.7)	197.3 (195.4, 199.2)	122.9 (121.3, 124.5)	45.8 (45.2, 46.4)	151.3 (144.7, 158.0)	93.9 (92.9, 95.0)	95.4 (94.7, 96.1)
Temuco, Chile	29.0 (28.8, 29.3)	96.5 (95.9, 97.1)	125.9 (125.1, 126.8)	81.1 (80.6, 81.7)	202.0 (200.0, 204.0)	125.6 (123.9, 127.2)	45.0 (44.4, 45.6)	169.1 (162.1, 176.2)	100.5 (99.1, 101.9)	99.7 (99.0, 100.3)
Barros Blancos, Uruguay	28.7 (28.4, 29.0)	98.8 ( 98.0, 99.6)	129.5 (128.5, 130.6)	81.6 (81.0, 82.2)	208.3 (206.0, 210.6)	133.0 (131.1, 135.0)	48.2 (47.6, 48.9)	140.3 (134.4, 146.1)	95.7 (94.4, 96.9)	95.4 (94.6, 96.2)

Age groups, year										
35-44	28.5 (28.2, 28.8)	94.5 (93.8, 95.2)	119.0 (118.2, 119.8)	80.2 (79.6, 80.8)	195.5 (193.3, 197.6)	122.4 (120.7, 124.2)	44.7 (44.1, 45.4)	151.7 (143.6, 159.8)	91.3 (90.4, 92.2)	106.9 (106.3, 107.5)
45-54	28.8 (28.5, 29.0)	96.1 (95.5, 96.8)	125.9 (125.0, 126.8)	83.3 (82.7, 83.9)	204.0 (201.9, 206.0)	128.1 (126.4, 129.9)	45.1 (44.5, 45.8)	165.7 (158.6, 172.8)	98.1 (96.6, 99.6)	98.8 (98.2, 99.4)
55-64	29.4 (29.2, 29.7)	98.7 (98.1, 99.4)	134.4 (133.5, 135.3)	84.4 (83.9, 85.0)	209.4 (207.3, 211.6)	130.9 (129.1, 132.7)	46.7 (46.0, 47.3)	167.3 (161.1, 173.5)	104.8 (102.8, 106.8)	89.5 (88.8, 90.1)
65-74	29.2 (29.0, 29.5)	99.3 (98.5, 100.0)	141.7 (140.6, 142.9)	83.0 (82.4, 83.6)	202.7 (200.2, 205.1)	125.2 (123.1, 127.3)	48.1 (47.4, 48.8)	149.9 (145.2, 154.7)	106.7 (104.4, 108.9)	81.5 (80.8, 82.2)

eGFR: estimated glomerular filtration rate was calculated using the CKD-EPI equation.

**Table 3:** Prevalence of Cardiometabolic Risk Factors in Southern Cone of Latin America

	Overweig ht	Obesit y	Centr al Obesit y	Hypertensi on	CK D	Hyper- cholesterole mia	High LDL- cholester ol	Low HDL- cholester ol	Hyper- triglyceride mia	Dyslipide mia	Diabet es	Metabol ic syndro me
Overall	41.3 (39.9, 42.7)	35.7 (34.4, 37.0)	52.9 (51.6, 54.3)	40.8 (39.4, 42.1)	2.0 (1.7, 2.3)	24.4 (23.3, 25.6)	23.1 (22.0, 24.3)	34.1 (32.8, 35.5)	22.1 (20.9, 23.3)	58.4 (57.0, 59.8)	12.4 (115, 13.3)	37.4 (36.0, 38.7)

Sex												
Men	47.7 (45.6, 49.8)	31.9 (30.0, 33.8)	35.7 (33.7, 37.7)	44.7 (42.6, 46.7)	1.8 ( 1.4, 2.2)	23.1 (21.4, 24.9)	21.9 (20.2, 23.6)	46.6 (44.5, 48.7)	29.6 (27.6, 31.5)	68.3 (66.3, 70.2)	10.6 ( 9.4, 11.7)	34.5 (32.5, 36.4)
Women	35.5 (33.7, 37.3)	39.1 (37.3, 40.9)	68.4 (66.6, 70.1)	37.3 (35.5, 39.0)	2.2 ( 1.7, 2.6)	25.6 (24.0, 27.1)	24.2 (22.6, 25.7)	22.9 (21.3, 24.6)	15.5 (14.1, 16.8)	49.6 (47.7, 51.5)	14.0 (12.8, 15.3)	40.0 (38.2, 41.8)

Location												
Marcos Paz, Argentina	34.2 (31.9, 36.6)	44.7 (42.3, 47.2)	54.4 (51.9, 56.8)	41.0 (38.7, 43.4)	2.3 ( 1.7, 2.9)	22.7 (20.7, 24.6)	21.2 (19.2, 23.2)	38.3 (35.8, 40.7)	23.2 (21.0, 25.4)	61.8 (59.4, 64.1)	11.9 (10.4, 13.4)	38.5 (36.2, 40.9)
Bariloche, Argentina	40.0 (37.7, 42.4)	32.2 (30.0, 34.4)	46.5 (44.1, 48.8)	45.3 (42.9, 47.6)	1.7 ( 1.2, 2.2)	20.8 (18.9, 22.6)	19.5 (17.7, 21.4)	32.7 (30.5, 35.0)	19.3 (17.4, 21.2)	53.6 (51.2, 55.9)	8.4 ( 7.2, 9.6)	35.5 (33.3, 37.7)
Temuco, Chile	45.5 (43.0, 47.9)	35.6 (33.3, 37.9)	54.9 (52.5, 57.3)	36.9 (34.6, 39.1)	1.7 ( 1.3, 2.2)	24.8 (22.8, 26.8)	22.9 (20.9, 24.9)	36.9 (34.5, 39.3)	25.7 (23.6, 27.9)	61.6 (59.2, 64.0)	14.3 (12.7, 15.8)	39.0 (36.6, 41.4)
Barros Blancos, Uruguay	35.1 (32.6, 37.6)	36.7 (34.2, 39.2)	57.6 (55.0, 60.2)	44.5 (41.9, 47.0)	3.2 (2.4, 3.9)	31.0 (28.7, 33.4)	31.6 (29.2, 34.1)	25.4 (23.1, 27.8)	15.7 (13.8, 17.6)	55.2 (52.6, 57.8)	14.2 (12.5, 15.9)	35.1 (32.6, 37.5)

Age groups, yr												
35-44	41.3 (38.6, 44.1)	32.4 (29.8, 35.0)	46.0 (43.2, 48.7)	22.5 (20.2, 24.8)	0.1 (0.0 , 0.3)	13.5 (11.5, 15.4)	12.4 (10.6, 14.3)	36.9 (34.1, 39.6)	20.4 (18.1, 22.7)	52.6 (49.8, 55.4)	6.1 ( 4.7, 7.4)	25.8 (23.3, 28.2)
45-54	42.1 (39.7, 44.6)	35.3 (33.0, 37.7)	51.7 (49.2, 54.1)	38.8 (36.4, 41.2)	0.7 ( 0.3, 1.1)	24.2 (22.1, 26.4)	23.6 (21.4, 25.7)	35.9 (33.5, 38.3)	23.4 (21.3, 25.6)	59.6 (57.1, 62.0)	11.1 ( 9.5, 12.7)	37.7 (35.3, 40.1)
55-64	41.2 (38.8, 43.6)	39.3 (37.0, 41.7)	60.2 (57.9, 62.6)	57.2 (54.8, 59.6)	3.5 ( 2.6, 4.3)	35.6 (33.3, 38.0)	33.5 (31.1, 35.9)	31.8 (29.5, 34.1)	25.0 (22.8, 27.2)	64.4 (62.0, 66.7)	18.4 (16.5, 20.3)	48.5 (46.1, 50.9)
65-74	39.3 (36.7, 42.0)	40.6 (37.9, 43.2)	64.6 (62.0, 67.2)	72.4 (69.9, 74.8)	8.2 ( 6.8, 9.7)	39.2 (36.5, 41.9)	36.8 (34.1, 39.5)	25.7 (23.3, 28.1)	19.6 (17.5, 21.8)	63.2 (60.6, 65.8)	24.1 (21.7, 26.4)	53.0 (50.3, 55.7)

Overweight: body-mass index ≥25 and <30 kg/m^2^; Obesity: body-mass index ≥30 kg/m^2^; Central obesity: waist circumference ≥102 for men and ≥88 cm for women; Hypertension: systolic blood pressure ≥140 mm Hg and/or diastolic blood pressure ≥90 mm Hg and/or use of antihypertensive medication; Chronic kidney disease: estimated-glomerular filtration rate <60 ml/min/173 m2; Hypercholesterolemia: total cholesterol ≥240 mg/dL and/or use of lipid-lowering medication; High LDL-cholesterol: LDL-cholesterol ≥160 mg/dL and/or use of lipid-lowering medication; Low HDL-cholesterol: HDL-cholesterol <40 mg/dL; Hypertriglyceridemia: triglyceride ≥200 mg/dL; Dyslipidemia: total cholesterol ≥240 mg/dL and/or LDL-cholesterol ≥160 mg/dL and/or HDL-cholesterol <40 mg/dL and/or triglyceride ≥200 mg/dL and/or use of lipid-lowering medication; Diabetes: fasting glucose ≥126 mg/dL or selfreported history of diabetes; Metabolic syndrome: 3 or more metabolic risk factors (waist circumference ≥102 cm in men and 88 cm in women, triglyceride ≥150 mg/dL, HDL-cholesterol <40 mg/dL in men and 50 mg/dL in women, blood pressure ≥135/85 mm Hg or use of antihypertensive medications, and fasting glucose ≥110 mg/dL or anti-diabetic therapy).

## Highlights

- Cardiovascular disease risk factors are highly prevalent in the general population in the Southern Cone of Latin America.
- The prevalences of obesity, hypertension, dyslipidemia, and diabetes were 35.7%, 40.8%, 58.4%, and 12.4%, respectively.
- The prevalence of metabolic syndrome was 37.4%.
- The proportion of individuals with ≥3 cardiovascular risk factors was 68.3%.

## Abbreviations

BMI: body-mass index
BP: blood pressure
CESCAS: Centro de Excelencia en Salud Cardiovascular para el Cono Sur
CHD: coronary heart disease
CKD: chronic kidney disease
CVD: cardiovascular disease
HDL: high-density lipoprotein
LDL: low-density lipoprotein
LMIC: low- and middle-income countries
MET: metabolic equivalent
